# Trend of vaccine preventable diseases in Iraq in time of conflict

**DOI:** 10.11604/pamj.2018.31.130.16394

**Published:** 2018-10-22

**Authors:** Riyadh Lafta, Ashraf Hussain

**Affiliations:** 1 AMustansiriya University, College of Medicine, Global Health Department, University of Washington, Seattle, USA; 2 University of Babil, College of Medicine, Babil, Iraq

**Keywords:** Trend, vaccine, preventable, diseases, Iraq, conflict

## Abstract

**Introduction:**

Iraq has passed through a series of successive conflicts, economic sanction and violence. The overall health sector in Iraq has been plunged and the services are facing a continuous shortage in vaccines, medicines and other supplies, and access of people to the basic health services being more impaired. The objective of this study was to portray the trend of vaccine preventable diseases in Iraq during the past 17 years to provide baseline information for disease burden estimation.

**Methods:**

This study was built on collection and treatment of morbidity data related to vaccine preventable diseases (tuberculosis, poliomyelitis, measles, mumps, rubella, diphtheria, tetanus, pertussis, and hepatitis B) that were registered by the Department of Health Statistics during the years (2000-2016). The incidence rates were plotted on a timeline to define the trend of each disease. Data were also categorized by gender and age groups (less than five years, 5 to 15 years and 15 years and more).

**Results:**

Diphtheria, rubella, and tuberculosis showed a slowly down going trend of incidence while mumps demonstrated a peak at 2016. Hepatitis B showed an up going trend of incidence while measles showed a secular trend every 4-5 years.

**Conclusion:**

Vaccine preventable diseases are still causing outbreaks; precipitated by fluctuation of vaccine coverage. Tuberculosis has been reemerged after a relatively long period of control.

## Introduction

National and local authorities need to set priorities for health needs and take decisions regarding investment in the health system while facing limited resources, ongoing raised demands for health care, developing new interventions and growing health care costs [1]. Burden of illness captures signals about the impact of diseases and the related risk factors that affect individuals, the government and the society as a whole [2]. Iraq is a high middle income developing country [3]. It had the most advanced health system in the Middle East during the 1960s through 1980s [4]. Depending on the revenues of oil industry, it ensured free health care in 172 hospitals and 1200 primary health center [5], and provided a good sanitary infrastructure and safe water supply to almost all urban and the majority of rural population [6]. In 1985, the Expanded Program of Immunization was established nationwide [7]. Iraq had been certified as polio free with almost controlled measles outbreaks [8]. But the escalation of fighting, associated with vast population displacement, resulted in fragmentation of the immunization program. Limited access to the health services resulted in an increased risk of outbreaks of infectious diseases and constrained the efforts to control them [9]. The national immunization coverage of measles declined to 75% instead of the recommended 95% [10], wild measles virus imported from Syria resulted in an outbreak in 2014 which followed an outbreak in 2008/2009 [7], vaccine failure became exceptionally high with a rate that reached up to 60.1% [11]. The unstable electric power all over Iraq, especially in the fighting zones, disrupted the cold chain for polio vaccine [12], which accompanied the replacement of the trivalent oral polio vaccine by the bivalent injectable one [13].

Despite the application of the national TB program since 1989 (followed by DOTS in 1998); Iraq still shows the highest TB rate in the region with about 15000 TB cases per annum [14]. The incidence in 2015 was about 43/100000 with a relatively low multi drug resistance (3.3/100000) that accounts for 1.1% of the new and relapsed TB cases [15, 16]. Contaminated water supply and poor sanitation remain important concerns where raw sewages are evacuated untreated in rivers and bodies of water [17, 18]. By MICS4 survey (2011), 91% of the urban populations and 77% of the rural households had access to safe drinking water, while those who do not, had no other facility for water treatment [19, 20]. This critical situation kept Iraq endemic with cholera which had repeated outbreaks in 2007, 2008, 2012, 2013 and 2015 that registered more than 2800 confirmed cases in 17 out of 18 governorates [21]. Human development 2016 report ranked Iraq at 121^st^ that is lower than most of the neighboring countries. UNDP report presented that in 2015 one fifth of the Iraqi population was under the poverty line. This was associated with relatively low health expenditure; been 3.3% of the total GDP. The overall health sector been plunged and the services are facing a continuous shortage in medicines and other supplies, and access of the people to the basic health services being more impaired [22]. Iraq is hyper-endemic in hepatitis A (96.4%) [9], while it is of low endemicity of both hepatitis B (1.6%) and C (0.4%) [21], with a national infection control strategy that all donated blood been screened for HBV and HCV [23]. There is no previous attempt in Iraq to find the trend of vaccine preventable diseases, so we set this study to portray an epidemiological trend of these diseases for the last 17 years to provide baseline information for disease burden estimation.

## Methods

This study was built on collection and treatment of morbidity data related to vaccine preventable diseases (tuberculosis, poliomyelitis, measles, mumps, rubella, diphtheria, tetanus, pertussis, and hepatitis B) that were registered by the Department of Health Statistics during the years (2000-2016). Data were gathered month by month, double-checked and confirmed by data from other official sources to enhance reliability. Data were categorized according to the 18 Iraqi governorates; the total of each governorate was obtained from the Central Statistical Organization (CSO) and summed to get the total for Iraq. The results were conformed to the reports of WHO, UNICEF, and the World Bank. The rates of occurrence were plotted versus time (for the period between 2000 through 2016) to define the trend of diseases. Data were also categorized by gender and age that was divided into three main categories: less than 5 years (the vulnerable category), 5 to less than 15 years, and 15 years and more that represent adults.

Data analysis: the statistical package for social sciences (SPSS version 23) was used to analyze the data. The trends present the rate of occurrence of diseases for the whole country distributed by age and sex. The ethical aspect was approved by the Ethical Committee in the College of Medicine, Mustansiriyah University.

## Results

**Figure 1:**
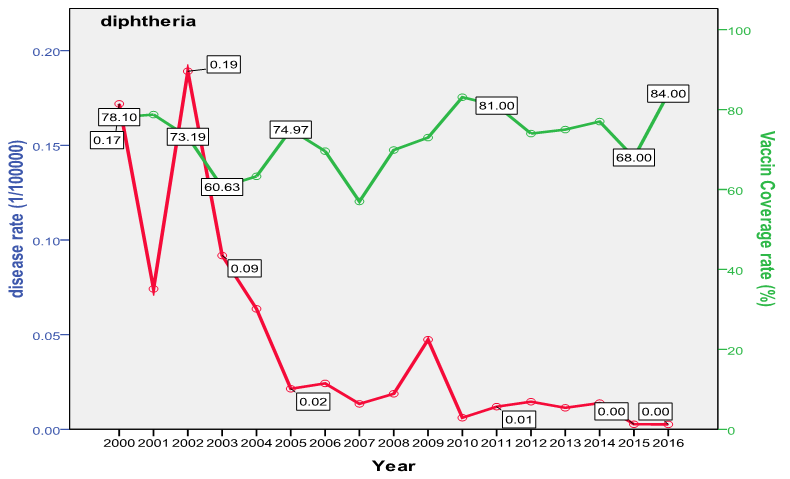
Trend of diphtheria in Iraq with vaccine coverage and age distribution

**Figure 2:**
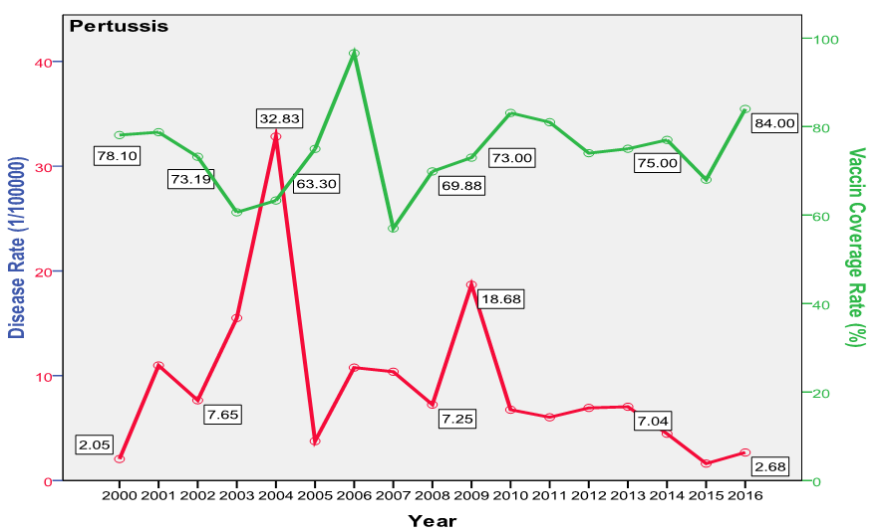
Trend of pertussis in Iraq with vaccine coverage and age distribution

**Figure 3:**
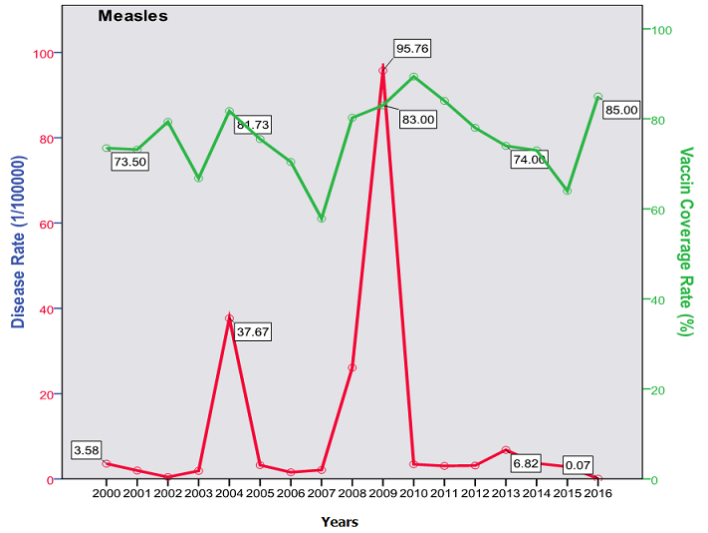
Trend of measles in Iraq with vaccine coverage rate

**Figure 4:**
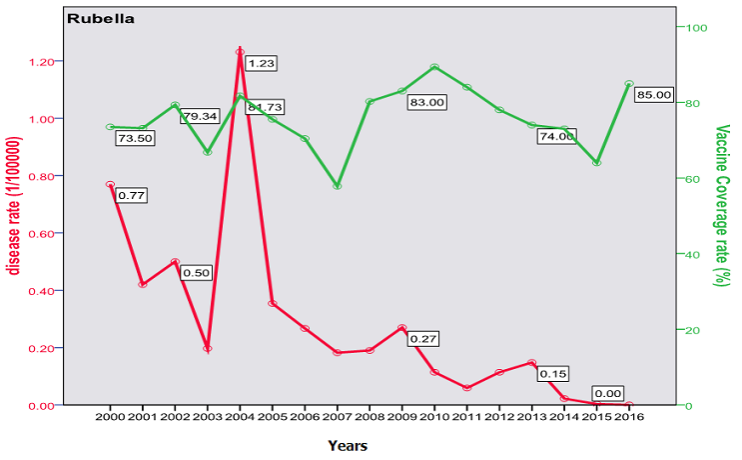
Trend of rubella in Iraq, vaccine coverage and age distribution

**Figure 5:**
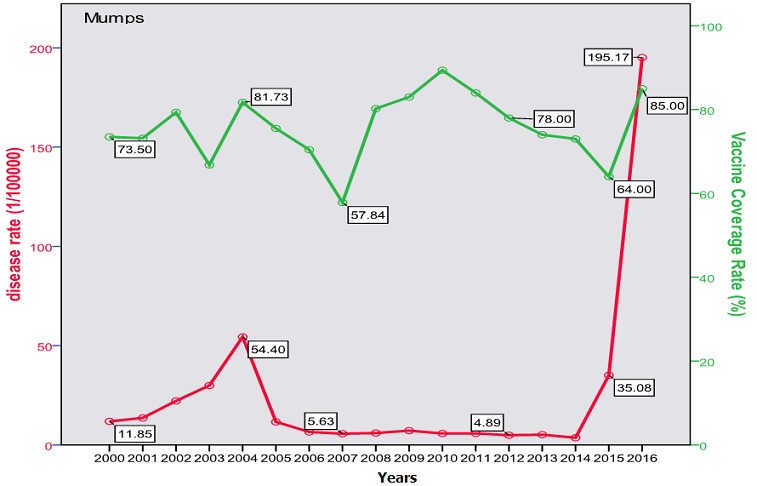
Trend of mumps in Iraq with vaccine coverage (2000-2016)

**Figure 6:**
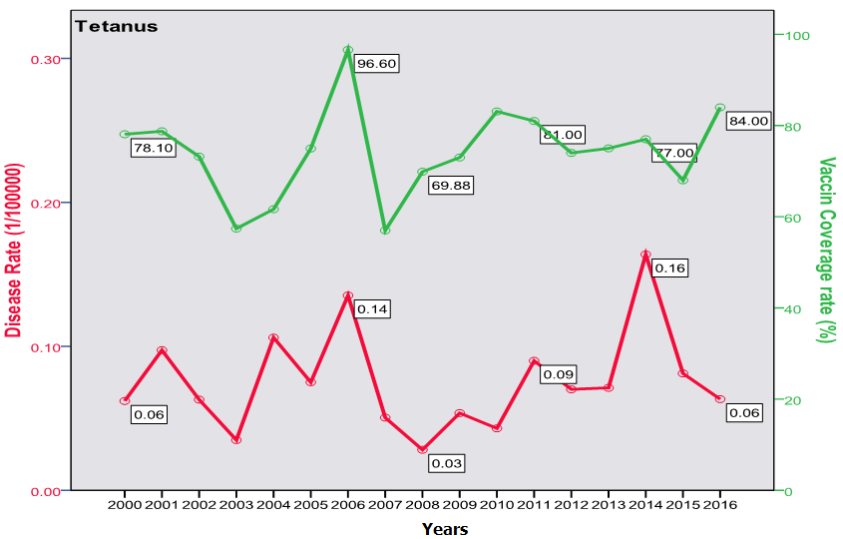
Trend of tetanus in Iraq with vaccine coverage (2000-2016)

**Figure 7:**
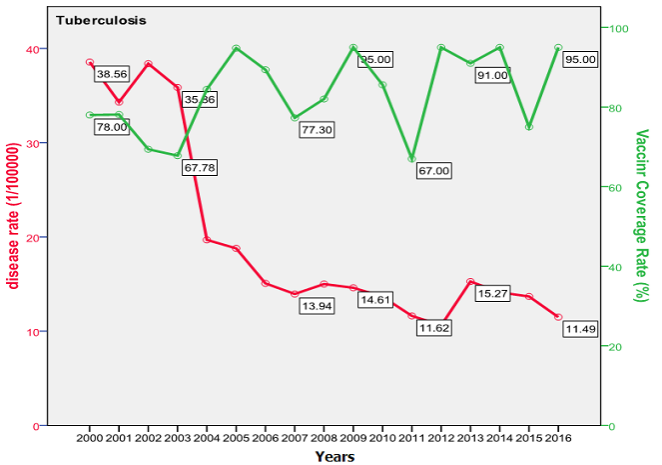
Trend of tuberculosis versus vaccine coverage (Iraq 2000-2016)

The trend of poliomyelitis incidence showed a long time of polio-free period until 2014 where a peak occurred. The peak consisted of two cases only; a male and a female, both in Baghdad. The trend of diphtheria incidence significantly went down over the studied period; from 0.172/100000 in the year 2000 to 0.003/100000 in 2016 (p=0.0003) as shown in Figure 1. The cases were unequally distributed between males and females and among the three age groups. The vaccine coverage rate was around 80 at that time. There was no significant change in the trend of pertussis (whooping cough) during the studied period. Both genders were almost equally affected; the most affected age group was children under 5 years with no shift in age of predilection. Vaccine coverage rate ranged between 70s and 80s in the last 10 years. Figure 2 shows the trend, vaccine coverage and age distribution of pertussis. Measles showed a secular trend with a peak every 4-5 years. No significant change in the trend over the surveyed period was detected (p=0.970). Males and females were equally affected. Vaccine coverage rate was fluctuating, and was below 70% in most of the years as shown in Figure 3. Figure 4 demonstrates the picture of rubella trend that shows a significant reduction in the rate over the studied years (p=0.002). Both genders were equally affected, mostly the age group (5-15 years). The trend of mumps showed a plateau for about 10 years (2005-2014), then a very high peak occurred in 2016 (p=0.220), with a male to female ratio of 3:2. The most affected age group was (5-15 years) (Figure 5). The trend of tetanus portrayed a zigzag pattern with non significant changes over time (p=0.586), affecting mostly males and those over 5 years of age (Figure 6). The trend of tuberculosis had significantly dropped within the studied period. It slowed down from 32.7/105 in 2000 to 9.66/105 in 2016 (p=0.0003), mostly among adult age group with an almost equal male to female ratio (Figure 7).

## Discussion

This study describes the trend of vaccine preventable diseases over 17 years that included years before and after the 2003 invasion of Iraq. The burden of these diseases comes from its high mortality particularly for the "less than five" age group, and the cost of treatment (especially antibiotics) considering the high incidence rates. Despite the involvement of measles vaccine in the Expanded Program of Immunization (EPI) since 1980 with two doses [24]; measles continued to occur at relatively higher rates than the neighboring countries. The mean rate of occurrence of measles in Iraq was 2.6/100000 (with exclusion of outbreak years: 2004, 2008-2009 and 2014), this could be considered high if compared to that of Jordan (0.16/100000), and Iran (0.09/100000) in 2014 [25]. Moreover, Iraq had witnessed three epidemics during the period from 2000 to 2016 that tend to occur almost every five years. The 2009 outbreak registered a rate of 95.7/100000. The findings of the current study reflect a high oscillation in measles vaccine coverage rates in Iraq during the period (2000-2016) which were between 70-85%, and sometime it was less than 60% [26]. There was an obvious increase in the incidence for each year that follows a drop in vaccine coverage; this was seen in most of the diseases in this study. The burden of measles is represented by its disabling and life threatening complications such as blindness, encephalitis, and measles associated pneumonia [27]. These serious complications usually occur in children age less than 5 years and may lead to death that may reach up to 10% [28]. A study in Iraq on 500 registered cases during the 2008-2009 outbreaks revealed a complication rate of 40%, pneumonia 33%, encephalitis 8/1000 and a case fatality rate of 2.5% [29]. The trend of mumps revealed a steady rate since 2004 of about 5/100000 (1500 to 2500 cases annually) influenced by the administration of MMR vaccine. This is still considered high if compared to that of Saudi Arabia or Jordan that registered less than 200 cases per year during the same period [30]. The trend showed an epidemic in 2004 with a rate of 54.4/100000 (13000 cases) and ended with an epidemic in the years 2015-2016 where it reached up to 195/100000 (74000 cases). Each of these epidemics followed a period of security instability that resulted in a disturbance of vaccination activities. Cases were mostly among age group 5-15 years with a slight predilection of males. Mumps is generally a mild childhood disease but can be associated with serious complications like meningitis (in 15% of cases), orchitis and 8^th^ cranial nerve involvement with deafness [31]. Rubella witnessed a significant decline after the last flare up in 2004 (300 cases with a rate of 1.23/100000) down to 7 cases in 2014 and zero case in 2016. This goes with the numbers in neighboring countries like Iran (23 case) and Jordan (only one case) [32]. Rubella is a mild childhood illness with a high risk on the susceptible pregnant that may lead to abortion or, more serious, congenital rubella syndrome with deafness, and eye, heart and brain defects. Unfortunately, no registered data is available regarding the occurrence of this syndrome in Iraq [33].

Poliomyelitis showed a zero rate all over the study period except in 2014 where two cases occurred, imported from Syria. Iraq was considered polio-free by WHO [34]. Tuberculosis trend showed a significant reduction in incidence from 32.74/100000 (8000 new cases) in the year 2000 down to 9.66 (4000 new cases) in 2016. This depression in the incidence was the result of the application of DOTS program since 1998 [35]. This trend is interesting when compared to some regional countries like Iran (32/100000 in year 2000 down to 22/100000 in 2014) [36] and Saudi Arabia (18/100000 down to 12/100000) [37]; but despite such progress, Iraq is still considered as one of seven regional high TB burden countries in the Eastern Mediterranean region. Tuberculosis treatment regimen puts the patient at a financial burden mainly due to loss of income. The health system in Iraq ensures free therapy which in turn cost about US$850 on average [38], this burden is positioned on the government rather than on the patient, further national burden comes from the lost days of work and premature death [39]. Diphtheria showed a significant reduction in rate of occurrence during the study period. The incidence dropped from 0.172 in the year 2000 to 0.003 in 2016 but with two peaks in 2002 and 2009 (42 and 15 cases respectively). This trend is similar to that of Saudi Arabia where the mean was 3 and lower than that of Iran (90) in the same period [40]. The main burden of diphtheria is the associated mortality of 5-10% which usually affects children less than 5 years and adults over 40 years [27]. Pertussis showed two peaks in 2004 and 2009 that occurred following a drop in vaccine coverage rate in the preceded years. After 2009, the disease showed a constant decrease in its occurrence during 2008-2016, with a mean occurrence of 2267 cases annually that is still very high if compared to Iran, Saudi Arabia and Jordan (600,7 and 3 respectively) [41]. The burden of pertussis is mainly due to its severity and the associated serious complications which necessitate hospitalization in 50% of the cases; these include bacterial pneumonia in 23%, seizure (1%) and encephalopathy (0.3%) and a case fatality rate that may reach up to 1%, mostly among infants [42].

The 2000-2016 trend of tetanus showed many peaks and drops swinging between 0.16 and 0.06. The mean number of cases was (23±12). In spite that this goes with that of the neighboring countries such as KSA (mean: 24/year) and Iran (19/year) for the same period, it still represents a burden because of its high case fatality rate even with the best intensive care facilities [43], most of our cases/deaths occurred prematurely, and predominantly below the age of 15 years. There was a down slop in the trend of incidence of Neonatal tetanus between the years 2000 and 2016. The rate of occurrence reduced from 0.18/105 (0.05/1000 live births) to 0.013/105 (0.005/1000 live births). Iraq was classified by WHO as one of the countries that eliminated neonatal tetanus [44]. Despite vaccination, the incidence of Hepatitis B disease showed no significant drop, on contrary, its rate rose from 4/100000 in the year 2000 up to 11.24/100000 in 2013 to slope down again reaching 5.2/100000 in 2016. Hepatitis C showed the same trend, it rose from 1.5/100000 in the year 2000 up to 3.61/100000 in 2011 and then started a zigzag, but the overall trend registered an increase in the incidence. In contrast, the global trend (2006-2016) of hepatitis B and C incidence witnessed an overall decline [45]. WHO attributed this rise to the improved diagnostic techniques that is the application of ELIZA at district level since 2013 which witnessed the drop [46]. The global cause specific mortality rate of hepatitis is 1.7/105; it is associated with progressive liver damage which ends eventually by premature death [47]. It is worth mentioning that there is a possibility of underestimation for most of the rates of the discussed diseases as many of our patients prefer to attend the private clinics and hospitals (for a better health care), so, they are not registered in the surveillance system of the Ministry of Health as it doesn't include private sectors.

## Conclusion

It can be concluded from this study that vaccine preventable diseases are still occurring in high incidence and causing outbreaks precipitated by fluctuation in vaccine coverage. Tuberculosis reemerged after a relatively long period of control.

### What is known about this topic

- Burden of illness captures signals about the impact of diseases and the related risk factors that affect individuals, governments and the society as a whole;
- The burden of vaccine preventable diseases comes from its high mortality particularly for the "less than five" age group;
- The overall health sector in Iraq been plunged and the services are facing a continuous shortage in medicines and other supplies.

### What this study adds

- Vaccine preventable diseases are still occurring in high incidence in Iraq and causing outbreaks precipitated by fluctuation in vaccine coverage;
- Urgent intervention from Ministry of Health and other supporting organizations is needed to prevent flare up of these diseases.

## Competing interests

The authors declare no competing interests.
